# Pulmonary Function and Clinical Manifestations of Patients Infected with Mild Influenza A Virus Subtype H1N1: A One-Year Follow-Up

**DOI:** 10.1371/journal.pone.0133698

**Published:** 2015-07-28

**Authors:** Wei Liu, Liping Peng, Hongmei Liu, Shucheng Hua

**Affiliations:** Department of Respiratory Medicine, First Hospital, Jilin University, Changchun, 130021, Jilin Province, China; The Scripps Research Institute, UNITED STATES

## Abstract

**Objective:**

To investigate the long-term effects of mild H1N1 influenza infection on the pulmonary function of a cohort of patients.

**Methods:**

Forty-eight patients, all diagnosed with influenza A virus subtype H1N1 in 2009, were retrospectively included in this study. Each patient in the study was monitored for 11-13 months by standard pulmonary function examination. The examination included monitoring respiratory tract infection symptoms (cough, expectoration or gasping) and vital signs. Long-term changes in symptoms and changes in vital signs were correlated back to and compared with the severity of the initial H1N1 influenza infection.

**Results:**

One year post discharge, mild to moderate pulmonary dysfunction was observed in the majority of patients. Further, 54.2% of patients had signs of severe abnormal pulmonary function, including diffusion disorder (33.3%) and small airway dysfunction (33.3%). Fourteen patients presented with respiratory tract infection symptoms; 12 with abnormal pulmonary function and two with normal pulmonary function. Our results indicated that the change in pulmonary function at one year post discharge was not significantly correlated with the severity of H1N1 influenza.

**Conclusion:**

Signs and symptoms of abnormal pulmonary function accompanied by respiratory tract infection symptoms remain for some patients after one year following discharge from the hospital for mild influenza A virus subtype H1N1 infection. These patients should continue to be monitored for any changes in condition and symptoms and rehabilitation treatment should be provided when necessary.

## Introduction

Influenza A virus subtype H1N1, a pandemic 2009 strain, caused widespread outbreaks of influenza in humans. As of 17 June 2010, more than 214 countries had reported confirmed cases of infection with pandemic 2009 influenza A (H1N1) virus [1]. Patients typically presented with severe pneumonia and acute respiratory distress syndrome (ARDS), which led to severe lung damage and in some cases death. After recovery from severe pneumonia and ARDS, various degrees of lung lesions occur, having an impact on patients’ respiratory function and in turn his or her quality of life. In this study, we examined the pulmonary function of patients infected with influenza A virus subtype H1N1 one year after hospitalization for the infection. These results provide valuable information for future diagnosis and rehabilitation treatment of H1N1 and other pandemic or severe influenza strain infections.

## Materials and Methods

### Subjects

A one-year pulmonary function follow up of was performed in 48 (48%, 26 men and 22 women) of the 102 (54 men and 48 women) patients diagnosed with mild influenza A virus subtype H1N1 at the First Hospital, Jilin University, China in 2009. Each patient was diagnosed by a physician according to the inclusion criteria of Influenza A Virus Subtype H1N1 Diagnosis and Treatment Protocol (Edition 3, 2009), issued by China’s Ministry of Health [2]. To ensure patients were not examined during or shortly after airway infections, all participants answered a questionnaire detailing any complaints of dyspnea, tiredness, cough, expectoration, medical treatment and smoking habits. The Modified Medical Research Council Dyspnea Scale was used to evaluate dyspnea of patients with abnormal pulmonary function (a score of 4 points, 2 cases; 3 points, 4 cases; 2 points, 14 cases;1 point, 4 cases; and 0 points, 2 cases) and with normal pulmonary function (a score of 4 points, 2 cases; 3 points, 2 cases; 2 points, 8 cases;1 point, 10 cases; and 0 points, 2 cases). Of these 48 patients, 38 were diagnosed by members of the Department of Respiratory Medicine and ten were diagnosed by members of the Department of Infection. The study included 26 male and 22 female patients with an average age of 29.5 years (range 27–39.5). Of the original 102 patients, eight (7.8%) had died: one from pneumonia and seven from disorders that could not be attributed to pulmonary disease. Forty-six (45.1%) patients were not re-examined due to practical problems. However, based on the data from 2009, these 46 patients did not differ from the 48 re-examined patients with respect to age, sex, disease duration, or degree of pulmonary function. Patients with chronic respiratory system disease (i.e. chronic obstructive pulmonary disease, asthma, pulmonary fibrosis, silicosis), chronic heart disease, or nervous and mental diseases were excluded. Written informed consent was obtained from each subject.

### Ethics statement

The experimental protocol was established, according to the ethical guidelines of the Helsinki Declaration and was approved by the Human Ethics Committee of Jilin University, China. Written informed consent was obtained from individual participants.

### Pulmonary function tests

Approximately one year (±1 months) after recovery from influenza and discharge from the hospital, each patient included in the study was assessed for pulmonary function using the MasterScreen PFT system (Jaeger, Germany). The indices for pulmonary function as part of this test include: Tidal volume (VT), vital capacity (VC), flow-volume loop, forced expiratory volume at 1 second (FEV1), maximal mid-expiratory flow (MMEF), forced expiratory flow at 50% and 75% (FEF50, FEF75) and maximum voluntary ventilation (MVV). The indices for pulmonary diffusion function include diffusing capacity of the lungs for carbon monoxide (DLCO) and diffusion rate. Patients rested for 30 minutes before testing, and tests were performed in duplicate for each patient with the higher of the two values being included in the study.

### Clinical evaluation

Respiratory tract infection symptoms (e.g. cough, expectoration or gasping), vital signs and pulse oxygen saturation (SpO2) were evaluated for each patient. Retrospective evaluation: Clinical data including respiratory tract infection symptoms and vital signs [3, 4], chest CT, blood gas analysis, mechanical ventilation, and the presence of secondary infection was also retrospectively analyzed at one year post hospital discharge. Results from the current clinical testing and from the retrospective analysis were correlated with the severity of H1N1 influenza infection. When subjects’ naïve pulmonary function tests were normal by routine blood test and physical examination at the one-year follow-up test, chest X-rays showed no significant change.

### Statistical analysis

Statistical analysis was performed using SPSS 17.0 (SPSS Inc., Chicago, IL, USA). *P* < 0.05 was considered statistically significant. Numeration data were expressed as the incidence rate, a chi-square test was used, data were expressed as mean ± standard deviation, and parametric statistics was used.

## Results

In order to assess the potential long term effects of mild H1N1 influenza infection patients were first assessed at approximately one year following recovery and hospital discharge. At this time, 29.2% (14/48) were observed to have obvious respiratory tract infection symptoms and 41.7% (20/48) had difficulties in performing physical activities. Pulse oxygen saturation was greater than 95% in all patients and no abnormal vital signs. We then tested each patient for pulmonary function and found 45.8% (22/48) had normal pulmonary function while 54.2% (26/48) had abnormal pulmonary function, all presenting with changes of mild to moderate H1N1 influenza. Several changes caused by abnormal pulmonary function were found, including diffusion disorder, small airway function disorder, and weakened storing function (Table 1).

**Table 1:** Change in pulmonary function at one year post-hospital discharge.

Change in pulmonary function	Incidence rate; n(%)
**Gas exchange function**	
Diffusion disorder (DLCO↓)	16/48 (33.3)
**Pulmonary ventilation**	
Small airway function disorder (FEF50↓, FEF75↓, MMEF75/25↓)	16/48 (33.3)
Simple limitation (TLC-He↓)	0 (0)
Mixed (small airway function disorder + simple limitation)	6/48 (12.5)
Weakened storing function (MVV↓)	14/48 (29.2)

DLCO: Diffusing capacity of the lungs for carbon monoxide; FEF50: forced expiratory flow at 50%; FEF75: forced expiratory flow at 75%; MMEF75/25: maximal mid-expiratory flow 75%-25%; MVV: maximum voluntary ventilation.

Of the 22 patients having normal pulmonary function, each had respiratory tract infection symptoms while six were observed to have a decreased ability to perform general physical activities. Of the 26 patients tested to have abnormal pulmonary function, 12 had respiratory tract infection systems and 14 had decreased ability to perform general physical activities. There was a clear correlation between respiratory tract infection symptoms and pulmonary function. Patients that tested for abnormal pulmonary function had a higher percentage respiratory tract infection symptoms when compared with the group of patients with normal pulmonary function (*P* = 0.047). Furthermore, patients with abnormal pulmonary function had a slightly, but not significant, greater influence on daily activities than normal pulmonary function (*P* = 0.188) (Table 2). Finally, ten patients were observed to have greater than three abnormal pulmonary function indices, manifesting as respiratory tract infection symptoms and resulting in decreased general physical activates.

**Table 2:** Correlation between pulmonary function one year post-hospital discharge and clinical manifestations of patients infected with influenza A virus subtype H1N1.

Clinical manifestations, n (%)	Normal pulmonary function (n = 22)	Abnormal pulmonary function (n = 26)	P value
Cough, expectoration or gasping	2 (9.1)	12 (46.2)	< 0.05
Influence on general physical activities	6 (27.3)	14 (53.8)	

Using the Modified Medical Research Council Dyspnea Scale, scores of four (two cases), three (four cases), two (14 cases), one (four cases) and zero (two cases) were observed for patients with abnormal pulmonary function. Similarly, for patients with normal pulmonary function, scores of four (two cases), three (two cases), two (eight cases), one (ten cases) and zero (two cases) were observed. There were no significant differences in pulmonary function and ARDS scores between patients with abnormal pulmonary function and patients with normal pulmonary function. In addition, there were no significant differences in total hospital days and poorest oxygenation index between patients with normal pulmonary function and patients with abnormal pulmonary function. Taken together, these result do not represent a correlation between pulmonary function at one year after discharge and the severity of the initial influenza infection (Table 3).

**Table 3:** Correlation between pulmonary function one year post-hospital discharge and hospitalization indices of patients infected with influenza A virus subtype H1N1.

Patients	n	Hospital duration (days)	Poorest oxygenation index
With normal pulmonary function	22	12.0±10.4	348.5±60.1
With abnormal pulmonary function	26	12.3±7.5	359.1±43.6
*P*		0.23	0.31

## Discussion

A common severe clinical manifestation of patients infected with influenza A virus subtype H1N1 is severe ARDS [5]. During recovery, pulmonary fibrosis is the major pathological change observed during recovery [4]. In addition, abnormal pulmonary function is manifested as decreased diffusion function and restrictive ventilatory disorder [6]. There is precedence for long term negative effects from pulmonary infection, as viral pneumonia-caused ARDS is a typical manifestation of severe acute respiratory syndrome (SARS) infections. Specifically, SARS patients presented with decreased pulmonary diffusion function during recovery [7–10]. Furthermore, a study by Neff et al [11] revealed that among 16 survivors of severe ARDS, 9 had abnormal pulmonary function, while four presented with obstructive ventilatory disorder and four with restrictive ventilatory disorder. In addition, a study by Li et al [12] found the incidence of obstructive ventilatory disorder and restrictive ventilatory disorder was approximately 30% following infection. Interestingly small airway dysfunction was also reported in a small number of SARS patients during recovery [8]. This is the first study to assess the long term effects of mild influenza A virus subtype H1N1.

Pulmonary diffusion disorder during H1N1 influenza infection recovery is similar to ARDS, however, a large proportion of patients recovering from influenza infection also show signs of small airway obstruction. In addition, this study reveals that approximately half of patients recovering from H1N1 influenza had abnormal pulmonary function, one third had diffusion dysfunction, a third had small airway obstruction, and another third presented with decreased ventilation function. The pathological changes following H1N1 influenza-induced severe pneumonia include three types: diffuse alveolar lesion, necrotizing bronchiolitis and widespread pulmonary hemorrhage [13]. This suggests that necrotizing bronchiolitis is likely to be the pathological basis of small airway obstruction. Here, we found 25% of patients had respiratory tract infection symptoms including cough, expectoration, or gasping, while 41.7% of patients had difficulties in performing general physical activities. Interestingly, the observed clinical symptoms correlated with patients having greater than three abnormal pulmonary function indices. In this study, we did not identify a relationship between abnormal pulmonary function of patients with H1N1 influenza and the severity of pulmonary function impairment during hospitalization, possibly because mild H1N1 influenza patients and a small number of H1N1 influenza patients were involved. This is consistent with previous studies investigating the effects of ARDS on pulmonary function [14, 15]. Several variables were not included in this study that may have also had an effect on the recovery of pulmonary function following influenza infection, including age, obesity, gender, recovery time, heart function, and the amount of physical rehabilitation exercise.

While some patients still have respiratory tract infection symptoms and limited physical activity one year after recovering from H1N1 infection. While no correlations were drawn between severity of infection and these symptoms, care should be paid to these patients, including follow-up pulmonary function tests to guide patients to the proper rehabilitation treatment, with the ultimate goal of improving patients’ quality of life.

## References

[1] Dawood FS, Jain S, Finelli L, Shaw MW, Lindstrom S, Garten RJ, et al Emergence of a novel swine-origin influenza A (H1N1) virus in humans. The New England journal of medicine. 2009;360(25):2605–15. Epub 2009/05/09. 10.1056/NEJMoa0903810 . 19423869

[2] Goka EA, Vallely PJ, Mutton KJ, Klapper PE. Mutations associated with severity of the pandemic influenza A(H1N1)pdm09 in humans: a systematic review and meta-analysis of epidemiological evidence. Arch Virol. 2014;159(12):3167–83. Epub 2014/08/01. 10.1007/s00705-014-2179-z . 25078388

[3] McMullen SM, Meade M, Rose L, Burns K, Mehta S, Doyle R, et al Partial ventilatory support modalities in acute lung injury and acute respiratory distress syndrome-a systematic review. PloS one. 2012;7(8):e40190 Epub 2012/08/24. 10.1371/journal.pone.0040190 ; PubMed Central PMCID: PMCPmc3420868. 22916094 PMC3420868

[4] Ranieri VM, Rubenfeld GD, Thompson BT, Ferguson ND, Caldwell E, Fan E, et al Acute respiratory distress syndrome: the Berlin Definition. Jama. 2012;307(23):2526–33. Epub 2012/07/17. 10.1001/jama.2012.5669 . 22797452

[5] Laszlo G. Standardisation of lung function testing: helpful guidance from the ATS/ERS Task Force. Thorax. 2006;61(9):744–6. Epub 2006/08/29. 10.1136/thx.2006.061648 ; PubMed Central PMCID: PMCPmc2117098. 16936234 PMC2117098

[6] Su MC, Hsieh YT, Wang YH, Lin AS, Chung YH, Lin MC. Exercise capacity and pulmonary function in hospital workers recovered from severe acute respiratory syndrome. Respiration; international review of thoracic diseases. 2007;74(5):511–6. Epub 2006/09/09. 10.1159/000095673 . 16960439

[7] Luyt CE, Combes A, Becquemin MH, Beigelman-Aubry C, Hatem S, Brun AL, et al Long-term outcomes of pandemic 2009 influenza A(H1N1)-associated severe ARDS. Chest. 2012;142(3):583–92. Epub 2012/09/06. 10.1378/chest.11-2196 . 22948576

[8] Herridge MS, Tansey CM, Matte A, Tomlinson G, Diaz-Granados N, Cooper A, et al Functional disability 5 years after acute respiratory distress syndrome. The New England journal of medicine. 2011;364(14):1293–304. Epub 2011/04/08. 10.1056/NEJMoa1011802 . 21470008

[9] Schmidt R, Markart P, Ruppert C, Wygrecka M, Kuchenbuch T, Walmrath D, et al Time-dependent changes in pulmonary surfactant function and composition in acute respiratory distress syndrome due to pneumonia or aspiration. Respiratory research. 2007;8:55 Epub 2007/07/31. 10.1186/1465-9921-8-55 ; PubMed Central PMCID: PMCPmc1950506. 17662121 PMC1950506

[10] Ngai JC, Ko FW, Ng SS, To KW, Tong M, Hui DS. The long-term impact of severe acute respiratory syndrome on pulmonary function, exercise capacity and health status. Respirology (Carlton, Vic). 2010;15(3):543–50. Epub 2010/03/27. 10.1111/j.1440-1843.2010.01720.x . 20337995 PMC7192220

[11] Neff TA, Stocker R, Frey HR, Stein S, Russi EW. Long-term assessment of lung function in survivors of severe ARDS. Chest. 2003;123(3):845–53. Epub 2003/03/12. . 12628887 10.1378/chest.123.3.845

[12] Rios FG, Estenssoro E, Villarejo F, Valentini R, Aguilar L, Pezzola D, et al Lung function and organ dysfunctions in 178 patients requiring mechanical ventilation during the 2009 influenza A (H1N1) pandemic. Critical care (London, England). 2011;15(4):R201 Epub 2011/08/19. 10.1186/cc10369 ; PubMed Central PMCID: PMCPmc3387643. 21849039 PMC3387643

[13] Mauad T, Hajjar LA, Callegari GD, da Silva LF, Schout D, Galas FR, et al Lung pathology in fatal novel human influenza A (H1N1) infection. Am J Respir Crit Care Med. 2010;181(1):72–9. Epub 2009/10/31. 10.1164/rccm.200909-1420OC . 19875682

[14] Peters JI, Bell RC, Prihoda TJ, Harris G, Andrews C, Johanson WG. Clinical determinants of abnormalities in pulmonary functions in survivors of the adult respiratory distress syndrome. The American review of respiratory disease. 1989;139(5):1163–8. Epub 1989/05/01. 10.1164/ajrccm/139.5.1163 . 2712444

[15] Suchyta MR, Elliott CG, Jensen RL, Crapo RO. Predicting the presence of pulmonary function impairment in adult respiratory distress syndrome survivors. Respiration; international review of thoracic diseases. 1993;60(2):103–8. Epub 1993/01/01. . 8341851 10.1159/000196182

